# Case report of a huge adrenal pseudocystic tumour with dopamine secretion: treatment paradigm from a very rare case

**DOI:** 10.1093/jscr/rjaf1092

**Published:** 2026-01-20

**Authors:** Konstantinos Isaakidis, Ioannis Rouvelas, Dimitrios Schizas, Neoklis Kritikos, Pasi Pengermä, Aristotelis Kechagias, Theodoros Michelakos

**Affiliations:** Department of Digestive and Endocrine Surgery, Metropolitan General Hospital, Athens, Greece; Department of Upper Abdominal Surgery, Center for Digestive Diseases, Karolinska University Hospital Huddinge and the Division of Surgery and Oncology, Department of Clinical Science, Intervention and Technology (CLINTEC), Karolinska Institutet, Stockholm, Sweden; Department of Propedeutic Surgery, “Laikon” General Hospital, National and Kapodistrian University of Athens, Athens, Greece; Department of Digestive and Endocrine Surgery, Metropolitan General Hospital, Athens, Greece; Department of Surgery, Mikkeli Central Hospital, Mikkeli, Finland; Department of Digestive and Endocrine Surgery, Metropolitan General Hospital, Athens, Greece; Division of Surgical Oncology Department of Surgery, Rush University Medical Center, Chicago, United States

**Keywords:** adrenal pseudocyst, dopamine secretion, secondary hypertension, posterior retroperitoneoscopic adrenalectomy, case report

## Abstract

Pheochromocytomas and extra-adrenal paragangliomas are rare neuroendocrine tumors typically characterized by excess secretion of epinephrine and/or norepinephrine. Dopamine secreting tumors are even more uncommon and predominantly extra-adrenal, with only a few solid adrenal ‘dopaminomas’ reported. To the best of our knowledge, we present the first case of a giant right adrenal pseudocystic tumor with autonomous dopamine secretion, incidentally discovered on computed tomography. The lesion was successfully excised via a 3-trocar posterior retroperitoneoscopic adrenalectomy, with an uneventful postoperative course. The patient, previously misdiagnosed with primary hypertension and sinus tachycardia, experienced complete resolution of cardiovascular symptoms postoperatively and discontinued antihypertensives and the b-blocker. Postoperative normalization of dopamine levels confirmed the tumor as the origin of the excess secretion. This case highlights the potential for dopamine-induced secondary hypertension and tachycardia, emphasizing the importance of including dopamine assays in the routine hormonal evaluation of adrenal masses, particularly when large or associated with hypertension and/or tachycardia.

## Introduction

Adrenal masses are classified as either adenomas with autonomous hormonal secretion or hormonally silent incidentalomas, which are discovered incidentally during imaging performed for unrelated medical reasons [1]. The adrenal hormonally active lesions have a variety of phenotypes and manifestations, with the most common being the corticosteroid secreting adenomas [1]. Pheochromocytomas, as well as extra-adrenal paragangliomas, are uncommon neuroendocrine tumors that over-secrete epinephrine and/or norepinephrine causing catecholamine-excess symptoms including hypertension, headache, palpitations, and sweating [1, 2]. A ‘dopaminoma’ is an extremely rare type of a large adrenal or extra-adrenal mass that secretes dopamine predominantly or exclusively, rather than norepinephrine or epinephrine [2, 3]. Another rare entity is the adrenal pseudocyst that is usually a benign and hormonally silent lesion, which is discovered incidentally during imaging [4]. Nonetheless, a limited number of adrenal cystic tumors with catecholamine secretion have been documented in literature [4].

To the best of our knowledge, we present the first case of a large right adrenal pseudocystic mass with autonomous dopamine secretion. Our aim is to provide insights into its pathophysiology and clinical management, and to propose refinements to the current hormonal diagnostic pathway recommended for adrenal incidentalomas [1]. This case report has been reported in line with the SCARE checklist [5].

## Case report

**Figure 1:**
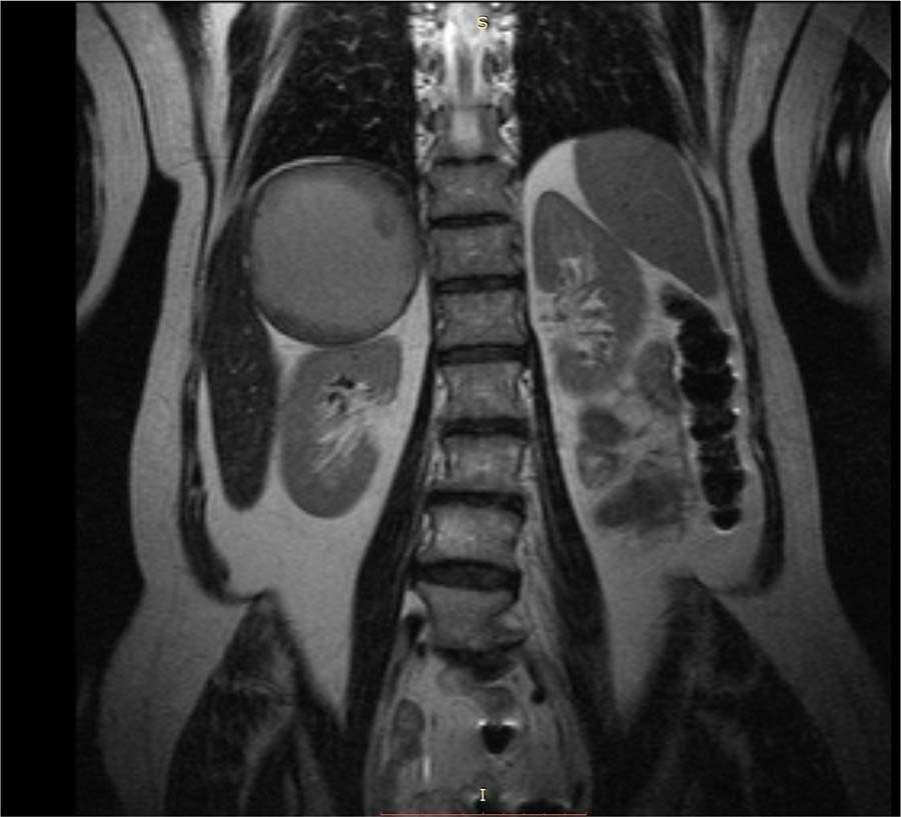
MRI caption showing a giant well-circumscribed right adrenal mass with solid components and thick fluid content. The remarkable dimension of the mass can be appreciated if compared to the right kidney.

A 60-year-old lady was referred to our department due to a giant right adrenal mass found incidentally on a computed-tomography (CT) scan performed three months earlier for pulmonary infection. The CT scan and magnetic resonance imaging (MRI) (Fig. 1) revealed an 11-centimeter right adrenal pseudocystic mass containing fluid and solid components, with parietal calcifications. Her medical history included anti-hypertensive medication (combination of angiotensin-II blocker and a calcium-channel blocker) and a b-blocker for increased sinus heart rate. From the complete hormonal laboratory only the plasma dopamine levels were increased in two separate samples (190.2 and 196.7 ng/L), raising the clinical suspicion of a dopamine-secreting mass.

**Figure 2:**
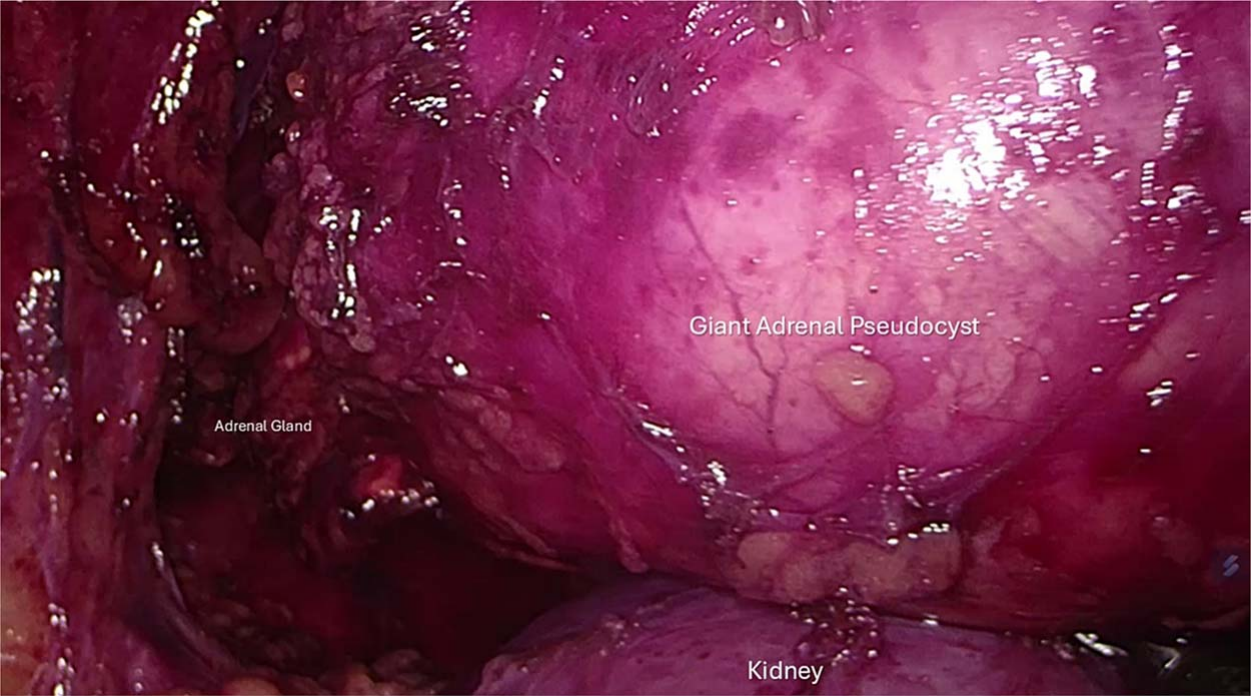
Intraoperative retroperitoneoscopic view showing the fully mobilized surgical specimen. The giant adrenal pseudocyst appears as a single, intact mass with the rest of the residual adrenal tissue attached medially. Due to its large size, the lesion cannot be entirely visualized within the operative field; its dimensions can be appreciated by comparison to the transverse diameter of the underlying right kidney.

The patient underwent the prone positioned 3-trocar posterior retroperitoneoscopic adrenalectomy (PRA). A huge cystic mass with solid components and a thick wall was confirmed with a size estimated at ~14 centimeters during retroperitoneoscopy. In Fig. 2 the right kidney is depicted as well for size comparison. The increase in size since the time of imaging diagnosis was attributed to the further accumulation of thick fluid component within the adrenal pseudocyst, however growth in the solid component could not be excluded. The mass was mobilized, completely removed, and extracted with a kidney-sized laparoscopic bag. The postoperative course was uneventful, and the patient was discharged 36 hours after surgery.

The patient experienced two episodes of mild hypotension within the first post-operative weeks, which were resolved at home. The postoperative cortisol levels were normal. Histology showed a benign adrenal pseudocystic mass of maximum dimension 14 centimeters, with areas of granulomatous inflammation. During the three postoperative months subsequent dopamine levels were found normal confirming the clinical diagnosis of a dopamine-secreting adrenal tumour. She gradually discontinued all previous medication as the blood pressure and the heart rate normalized naturally. At 3.5 years of clinical, radiologic, and laboratory follow-up there were no signs of disease recurrence.

## Discussion

Adrenal pseudocystic tumours are very rare and, to the best of our knowledge, this is the first case to be reported with hormonal hyperactivity due to dopamine-secretion. In general, dopamine secretion has been described almost exclusively from extra-adrenal masses such as paragangliomas or even from ganglioneuromas, which also constitute an uncommon entity [2, 6]. Dopamine secretion from solid non-pseudocystic adenomas localized within the adrenals is also extremely rare and described only in a few case reports [2, 3, 6, 7]. Unlike classic pheochromocytomas, dopamine-secreting adrenal adenomas lack significant dopamine β-hydroxylase activity, the enzyme that converts dopamine to norepinephrine [7]. Routine screening using blood and urinary metanephrines alone will not detect the excess of dopamine, therefore it is possible that the real number of tumors that secrete dopamine exclusively may be underestimated or misdiagnosed as a silent tumor [3]. Moreover, the symptomatology of a dopaminoma is still obscure and understudied due to the limited number of reported cases. Patients with a dopamine-secreting mass may be normotensive and lack catecholamine-excess symptoms (headaches, sweating, and palpitations) [6, 7]. In theory, there could be mild hypotension due to the dopamine’s vasodilatory effect, weight loss, nausea, and fatigue [2], however most reported cases did not present with any specific symptom or mass effect [3, 6, 7]. Consistently higher levels of dopamine have been documented to cause hypertension and tachycardia [7], as in our case.

The patient initially presented with a large incidentally discovered adrenal mass. Prior to the adrenal mass diagnosis, she had been misclassified as having primary hypertension and idiopathic sinus tachycardia. Following complete surgical resection of the adrenal lesion, there was a normalization of plasma dopamine levels accompanied by full resolution of cardiovascular symptoms. These findings confirmed the preoperative suspicion that the adrenal pseudocystic tumour was the source of autonomous dopamine secretion. Postoperatively, the patient discontinued all antihypertensive and b-blocker medications with sustained normalization of blood pressure and heart rate, while there were no signs of disease recurrence during long-term follow-up. This case represents secondary hypertension and tachycardia driven by dopamine excess from a large pseudocystic adrenal mass, successfully treated with the minimally-invasive PRA technique which offered quick postoperative recovery. The 3-trocar PRA is feasible for the resection of very large adrenal masses in high-expertise centers, although recommendations advocate even open surgery for such cases [1]. Current guidelines for the management of adrenal adenomas should be reassessed as they do not advocate the routine measurement of plasma or urinary dopamine, possibly resulting in underdiagnosis of dopamine-secreting tumors [1]. Moreover, the management of this case confirms that pre-operative administration of a1-blockers is unnecessary in the case of a dopaminoma [3], and that close postoperative blood pressure monitoring is required for the possibility of hypotension episodes.

## Conclusion

We report the first documented case of a giant dopamine-secreting adrenal pseudocystic tumor presenting with secondary hypertension and sinus tachycardia, both of which resolved completely following surgical excision. This case underscores the importance of including dopamine measurements in the routine hormonal evaluation of large adrenal masses or in the case of hypertension.

## Consent for publication

Written informed consent was obtained from the patient for publication of this case report and accompanying images. A copy of the written consent is available for review by the Editor-in-Chief of this journal on request.
